# *In situ* characterization of advanced glycation end products (AGEs) in collagen and model extracellular matrix by solid state NMR†
†Electronic supplementary information (ESI) available. See DOI: 10.1039/c7cc06624d


**DOI:** 10.1039/c7cc06624d

**Published:** 2017-12-01

**Authors:** R. Li, R. Rajan, W. C. V. Wong, D. G. Reid, M. J. Duer, V. J. Somovilla, N. Martinez-Saez, G. J. L. Bernardes, R. Hayward, C. M. Shanahan

**Affiliations:** a Department of Chemistry , University of Cambridge , Lensfield Road , Cambridge CB2 1EW , UK . Email: mjd13@cam.ac.uk ; Fax: +44(0)1223-336362 ; Tel: +44(0)1223-736394; b BHF Centre of Research Excellence , Cardiovascular Division , King's College London , London SE5 9NU , UK

## Abstract

Non-enzymatic glycation of extracellular matrix with (U-^13^C_5_)-d-ribose-5-phosphate (R5P), enables *in situ* 2D ssNMR identification of many deleterious protein modifications and crosslinks, including previously unreported oxalamido and hemiaminal (CH_3_–CH(OH)NHR) substructures. Changes in charged residue proportions and distribution may be as important as crosslinking in provoking and understanding harmful tissue changes.

## Abstract 2

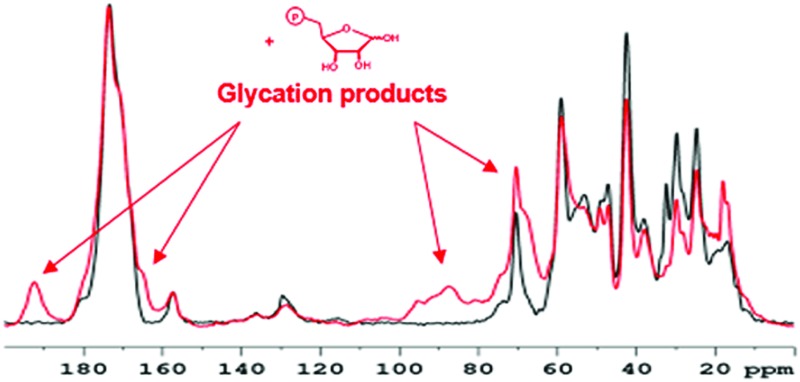
Pathological glycation of extracellular matrix modelled with ^13^C-labelled sugars yields unique novel atomic level NMR structural and chemical insights non-destructively.

## 

Glycation refers to spontaneously occurring non-enzymatic reactions between biogenic aldehydes and ketones, in particular the uncyclized forms of sugars, and biomolecular nucleophiles, importantly the amino and guanidino side chains of protein Lys and Arg.^1^ Glycation is initiated by Schiff base formation followed by Amadori rearrangement and thereafter a cascade of diverse and much studied but still incompletely characterized so-called “Maillard” reactions. These can lead to adduction of basic groups by acidic sugar glycoxidative breakdown products and thus changes in protein structure, net charge and charge distribution, and a variety of unnatural cross links between nearby residues. The effects of glycation are generally deleterious and result in altered tissue mechanical properties such as stiffness and tensile strength,^2^–^4^ net charge and charge distribution, and protein binding recognition sites,^5^ resulting in altered molecular^6^,^7^ and cellular^8^ recognition, and triggering of inflammatory processes *via* the “receptor of advanced glycation end products”, RAGE.^9^ Predictably glycation is more severe in hyperglycemic states such as diabetes, and affects slow turnover macromolecules, such as collagen, particularly markedly. Indeed glycation of connective tissues, especially blood vessels constantly exposed to high levels of circulating sugars, is a major factor underlying pathologies of diabetes, as well as normal ageing.^11^–^13^ It is thus of great biomedical importance to continue the characterization of glycation processes leading to advanced glycation end products (AGEs) and their effects on macroscopic and microscopic tissue properties, and initiate an understanding of the effects of glycation on biomolecular structure at the atomic level.

The complexity of the glycation process is compounded by its proceeding with a distinct lack of consistency under apparently consistent conditions, rendering pathways and structural consequences of glycation extremely difficult to study systematically *in vivo*. This complexity is exacerbated by the occurrence of reactions which are hard to predict even with simple model compounds,^14^ and the poorly understood catalytic influence of certain biomolecules and ions.^15^

Collagen is a major protein component of all vertebrate tissues and the principle constituent of the extracellular matrix (ECM) of connective tissue of bone, blood vessels, and numerous other organs. Apart from the mechanical and structural roles of the different connective tissue collagen isoforms, they all perform vital cell adhesion, motility, and signalling, roles the importance of which is being increasingly appreciated.^16^ The collagens consist of repeating triplet amino acid motifs –(Gly–X–Y)– in which X and Y are frequently Pro and Hyp respectively, imposing a unique triple helical secondary structure on the three polypeptide chains, which comprises the fundamental building block of collagenous tissue, including vascular smooth muscle (VSM) ECM. Collagen I triple helices self-associate into larger scale fibrillary structures, and additionally undergo a variety of orderly enzymatic post translational modifications leading to glycosylations and cross linking at specific residues.^17^

We have developed high yield methods of producing biomimetic collagenous VSM cell (VSMC) ECM reproducibly *in vitro*. This enables NMR active nuclei to be introduced into specific amino acids, and studying of their structural environment, dynamics, and chemistry, using powerful 2D (and potentially multidimensional) solid state NMR methods impossible with unlabelled materials. This provides a cost effective, and ethical, alternative to *in vivo* methods for producing sufficient material for NMR. Equally importantly it facilitates the incorporation of specific amino acids, and sugars, (and in some cases, unavoidably, their labelled metabolites) into matrix proteins for NMR structural studies. We have used fetal sheep osteoblast (FSOb) ECM (FSOb-ECM) enriched in U-^13^C,^15^N-labelled Gly and Pro, particularly prevalent in collagen, to validate our use of *in vitro* material against labelled native bone.^18^ By incorporating specific labelled amino acids into *in vitro* ECM it is possible to address detailed aspects of collagen and ECM molecular structure and how this changes under glycation. Ultimately by glycating matrix labelled with glycation target residues Lys, and Arg, with labelled sugar reagents, identification of covalent AGE structures *in situ* may be possible by ssNMR using similar atomic proximity-sensitive techniques to those described below, without recourse to potentially destructive degradative analyses.

A first step in this process requires the identification of those NMR signals which arise from labelled glycating agent, and which from labelled glycated protein. Accordingly this communication reports 2D NMR characterization of labelled products arising from reacting unlabelled native pure collagen, and unlabelled *in vitro* VSMC ECM, with U-^13^C labelled d-ribose-5-phosphate (U-^13^C_5_-R5P), an important intermediate in nucleic acid and energy metabolism,^19^,^20^ and a vigorous endogenous glycating agent.^21^,^22^

Materials and methods, including the synthesis of the sodium salt of U-^13^C_5_-R5P from commercial U-^13^C_5_-ribose, are described in detail in ESI.†

Fig. 1 compares the 1D ^13^C CP-MAS spectra of pure collagen and VSMC ECM before, and after, incubation with U-^13^C_5_-R5P. There are close similarities between the spectra of the two unreacted materials (Fig. S1a, ESI†), in particular the prominent signal at *ca.* 70 ppm from the γ-carbon atoms of Hyp, an effective NMR marker of collagenous tissue. It demonstrates that collagen is the major component of the *in vitro* material, with minor components of other integral ECM proteins, and lipid. Incubation with U-^13^C_5_-R5P results in the appearance of a number of new glycation product signals, which are qualitatively quite similar for the two materials although the extent of production of each is in many cases rather different (Fig. S1b, ESI†). The generation of new glycation product signals is clearly shown by a comparison of the spectra of the glycated materials with those of pure collagen, and of R5P, which are overlaid in Fig. S1c (ESI†). In order to assign these NMR signals, we have used 2D single quantum–double quantum (SQ–DQ) and proton driven spin diffusion (PDSD) correlation techniques.

**Fig. 1:**
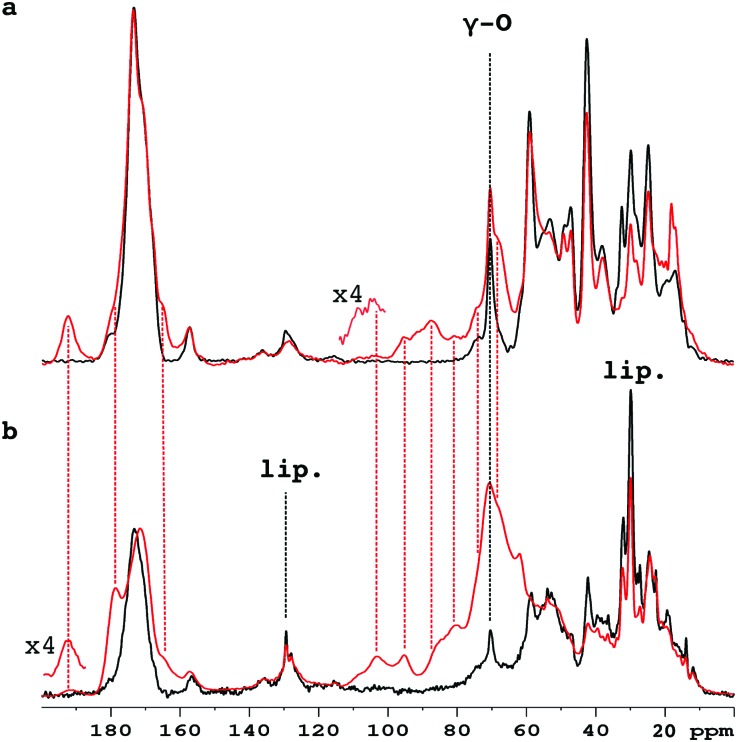
1D spectra of unlabelled collagen (a), and VSMC ECM (b) before (black), and after (red), incubation with U-^13^C_5_-R5P. The Hyp γ-carbon signal unique to collagen is labelled γ-O. The VSMC ECM material also shows signals at *ca.* 30 ppm and *ca.* 130 ppm from cross-polarizing lipid (lip.).

Accordingly Fig. 2 compares 2D SQ–DQ spectra of pure collagen, and of VSMC ECM, reacted with U-^13^C_5_-R5P. As the method depends on direct transfer mediated by the comparatively weak ^13^C–^13^C dipolar interaction, cross peaks effectively imply that the corresponding signals are from mutually bonded ^13^C atoms and therefore must originate from U-^13^C_5_-R5P. These connectivities are also probed by the PDSD experiment, which reveals longer range proximities as well depending on the experimental spin diffusion time. PDSD datasets, which essentially corroborate the SQ–DQ data, for the two glycated materials are compared in Fig. S2 (ESI†). Assignments are shown in Table 1, based in part on published data for a number of common glycation products (CEL,^23^ pentosinane, DOGDIC, and DOPDIC,^24^ glucosepane, GODIC, MODIC, DOGDIC, GOLD, and MOLD,^25^ and CML^26^) and strong cross peaks due to polyhydroxylated structures. A cross peak attributable to a putative 5-phospho-ribuloselysine, analogous to the initial ribuloselysine Amadori rearrangement product of ^13^C_5_-ribose, is not observed because the 5-phosphate group precludes cyclization to a stable furanose structure so this intermediate progresses rapidly to more advanced products.^21^ Clear cross peaks in SQ–DQ spectra of both glycated materials indicate the formation of a labelled fragment with two mutually bonded carbon atoms with near equal chemical shifts of *ca.* 165 ppm which we assign as part of an oxalate-derived structure. A distinct SQ–DQ correlation in U-^13^C_5_-R5P glycated collagen (not seen in VSMC ECM) between bonded carbons at shifts of 22 ppm and 91 ppm is consistent with a hemiaminal AGE substructure CH_3_CH(OH)NHR resulting from *e.g.* reaction of acetaldehyde with Lys. In general the products of the reaction between collagen and U-^13^C_5_-R5P qualitatively reproduce those observed from reaction with U-^13^C_5_-ribose.^10^ Although many AGE structures observed by NMR are common to both pure collagen and *in vitro* ECM there are quantitative differences, most likely due to differences in overall protein composition, and the accessibility of reactive groups to the glycating agents. The *in vitro* ECM probably more closely replicates an *in vivo* scenario as it would exist in the vasculature of a hyperglycemic patient for instance, in containing a variety of other integral ECM proteins besides type I collagen.

**Fig. 2:**
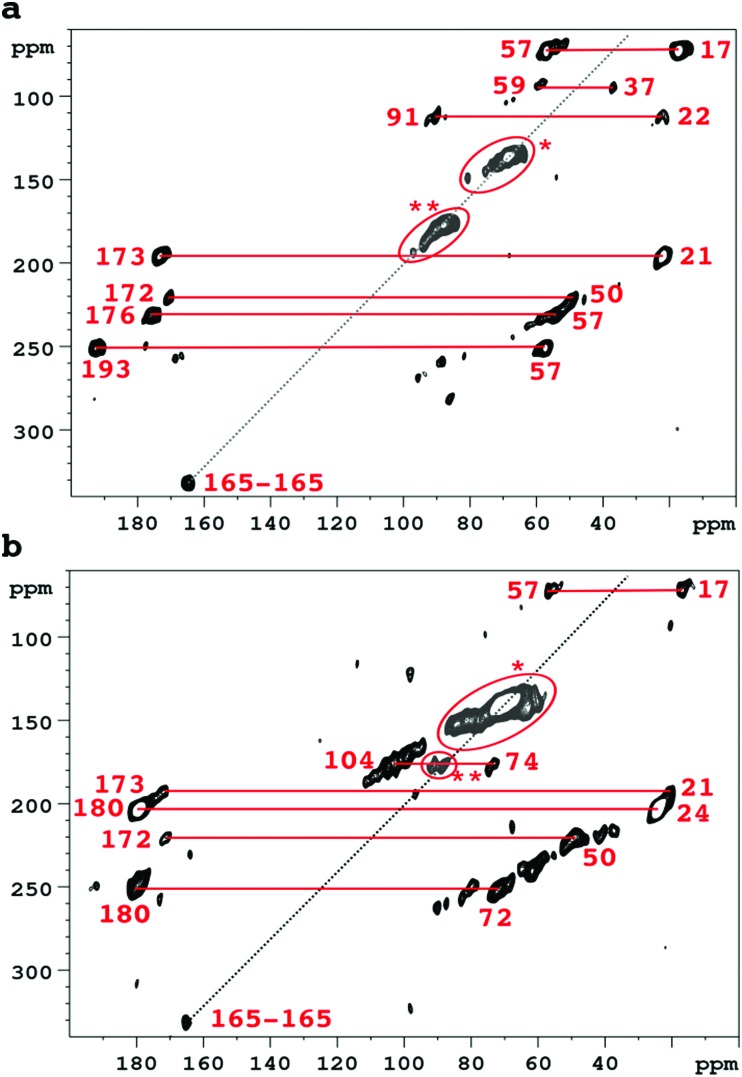
SQ–DQ correlation spectra of collagen (a), and VSMC ECM (b), after incubation with U-^13^C_5_-R5P. Correlations between significant glycation products are marked in red; correlations between carbon atoms with identical or very similar shifts are indicated *(vicinal diols) and **(hemiacetals, hemiaminal functionalities). The diagonal line represents the SQ–DQ axis.

**Table 1:** Summary of the ^13^C–^13^C SQ–DQ and PDSD (100 ms spin diffusion mixing time) connectivities in pure collagen, and VSMC ECM, reacted with ^13^C_5_-R5P, and comparison with products previously identified in pure collagen reacted with (U-^13^C_5_)-ribose^10^

Cross peak correlations		Assignment	VSMC	Collagen	U-^13^C_5_-ribose collagen^10^
Signal 1/ppm	Signal 2/ppm				
17	57	CEL and/or MODIC	Yes	Yes	Yes
17	193	Norpronyl lysine	Not obsd.	Yes	Not obsd.
21	173	*N*-Acetyls	Yes	Yes	Yes
24	180	*N*-Acetyls	Yes	Not obsd.	
22	91	CH_3_–CH(OH)NHR?	Not obsd.	Yes	Not obsd.
37	59	DOPDIC/pentosinane	Not obsd.	Yes	Yes
37	69	DOPDIC	Weak	Yes	Not obsd.
50	172	CML	Yes	Yes	Yes
57	176	CEL	V. wk.	Yes	Yes
57	193	Norpronyl lysine	Weak	Yes	Yes
62–84	62–84	Vicinal di-ols	Yes	Yes	Yes
74	104	Hydroxylic-(hemi)acetal carbons	Yes	Yes	Yes
64–73	178	DOGDIC, DOPDIC, MODIC, GODIC	Yes	Not certain	Yes
88–92	*Ca.* 90	(Hemi)acetal/aminal	Yes	Yes	Yes
165	165	Oxalic acid skeleton?	Yes	Yes	Not obsd.

Glycation is usually quantified using the natural fluorescence of specific AGEs,^27^ and antibody probes raised against others.^28^ Such methods obviously depend on the AGEs of interest being fluorescent in the first place, and the antigenicity of specific already-known AGE structures or non-specific structures resulting from glycation of an immunogenic protein, clearly leaving potential gaps in the AGE detection armory. Besides this many characterization approaches, for instance hyphenated chromatography–mass spectrometry, rely on hydrolysis of insoluble proteins such as collagen to constituent glycation-modified amino acids under rather severe conditions such as high temperatures and acidity; successful detection of certain AGEs thus clearly depends on their stability under these conditions. While NMR is considerably less sensitive than the above techniques it possesses the unique advantage that it can be applied to native glycated material with negligible pre-treatment and consequent possible decomposition, while the use of non-perturbing isotope labelled glycating agents is straightforward and greatly increases the atomic level information content of resulting data. Moreover our approach is directly applicable to other native biomaterials such as, importantly, bone,^29^,^30^ and *in vitro* model ECM.

It is widely assumed that changes in the mechanical and consequently biological properties of ECM are due mainly to the introduction of AGE induced crosslinks. While signals consistent with some cross linking structures (pentosinane, DOGDIC, DOPDIC, MODIC, GODIC) are observed in our materials, our data suggests that the most abundant AGEs formed are rather single amino acid residue modifications (CML, CEL, *N*-acetyl species), and nitrogen adducts of single ribosyl (phosphate) units. Such modifications convert basic protein functional groups into charge neutral (*N*-acetyl, *N*-sugar adducts) or negative (CML, CEL) substituents, with likely profound consequences for collagen triple helical structure, interfibril associations, hydration, and molecular recognition processes. Our results suggest that non-crosslinking, monovalent glycation products may be at least as important as AGE crosslinks in modifying ECM mechanical and molecular recognition properties.

Dr Jonathan Clark of the Babraham Research Institute for many helpful discussions; funding from the U.K. MRC (for DGR, RR, RH), Royal Society (URF) and FCT Portugal (iFCT) (both for GJLB), and for PhD studentships the China Scholarship Council and Cambridge Trust (for RL), and U.K. EPSRC (for VWCW).

## Conflicts of interest

There are no conflicts to declare.

## Supplementary Material

Supplementary information
