# Supplementary material for: In situ characterization of advanced glycation end products (AGEs) in collagen and model extracellular matrix by solid state NMR
Source: Chem Commun (Camb). 2017 Dec 1;53(100):13316–9. doi: 10.1039/c7cc06624d (PMC5774432; doi:10.1039/c7cc06624d)
Supplement: Supplementary file 1 [file CC-053-C7CC06624D-s001.pdf]

Electronic Supplementary Material (ESI) for ChemComm.
This journal is © The Royal Society of Chemistry 2017

Online Supplementary Material

In situ characterization of advanced glycation end products (AGEs) in collagen and model extracellular matrix by solid state NMR

Rui Li, Rakesh Rajan, Wai Ching Veronica Wong, David G. Reid, Melinda J. Duer*, Victor J. Somovilla, Nuria Martinez-Saez, Gonçalo J. L. Bernardes, Robert Hayward, Catherine M. Shanahan.

P. 1 Materials and Methods

P. 3 Synthesis of D-(U-$^{13}C_5$) - R5P

P. 6 Solid state NMR.

P. 7 Figure S1: Comparison of NMR of collagen and VSMC ECM before (a) and after (b) D-(U-$^{13}C_5$) - R5P incubation, and of native collagen and pure unreacted R5P.

P. 8 Figure S2: PDSD spectra of collagen (a) and VSMC ECM (b), after D-(U-$^{13}C_5$) - R5P incubation.

P. 9 References.

Materials and Methods

U-$^{13}C_5$ ribose was purchased from Cambridge Isotope Laboratories (Andover, MS USA) and bovine Achilles tendon type I collagen was from Sigma.

VSMCs were harvested from fresh adult bovine aortae. Cell culture reagents, media, suppliers, harvesting of VSMCs from bovine aortae, and their use in preparation of ECM, basically follows the methods used for fetal sheep osteoblast ECM described in detail in the supplementary material for Chow et al[1]. VSMCs were released from aortic strips by collagenase treatment and cultured to confluence in basal minimum Eagle's (BME) complete medium. Incubation was at 37°C in humidified 95% air, 5% CO_2. Media were renewed every 2 days until the cells and matrix began to detach from the culture flask, generally after ca. 9 days, by which time enough ECM had formed for SSNMR. Cells were washed with phosphate buffered saline (PBS). After 24 hours at -80ºC the cells were freeze-thaw lysed, debris removed by repeated PBS washes, and decellularized ECM dislodged by swirling the flask in PBS, centrifuged at (1200 rpm, 5 min, room temperature) and stored at -20°C until incubation with D-(U-$^{13}C_5$) - R5P.

Dry collagen was rehydrated overnight in aqueous acetic acid (10%), plunger homogenized, and washed with distilled water and centrifuged repetitively until no longer acidic, and stored wet at 4 °C until used. Bovine collagen, and bovine VSMC ECM, were incubated in 6 mL of (U-$^{13}C_5$)-ribose-5-phosphate (50 mM; see next section for synthesis) in 50mM sodium phosphate buffer at pH 7.4 and 37 ºC for 11 weeks, comparably to incubations with U-$^{13}C_5$-

ribose reported in Bullock et al[2].. Solution-state ^{13}C NMR spectra were recorded on the supernatant periodically to monitor glycation progress by the generation of small soluble glycation products. After incubation both glycated collagen, and glycated ECM, were washed several times with deionised water to remove unreacted sugar, phosphate, and soluble glycation products, freeze dried, and packed into a Bruker 4 mm o.d. zirconia MAS rotor (glycated collagen), or a Kel-F disposable insert (VSMC ECM) and stored at -20 ºC until ssNMR analysis.

Synthesis of D-(U-$^{13}C_5$) - R5P from U-$^{13}C_5$-D-ribose was carried out according to the following reaction scheme:

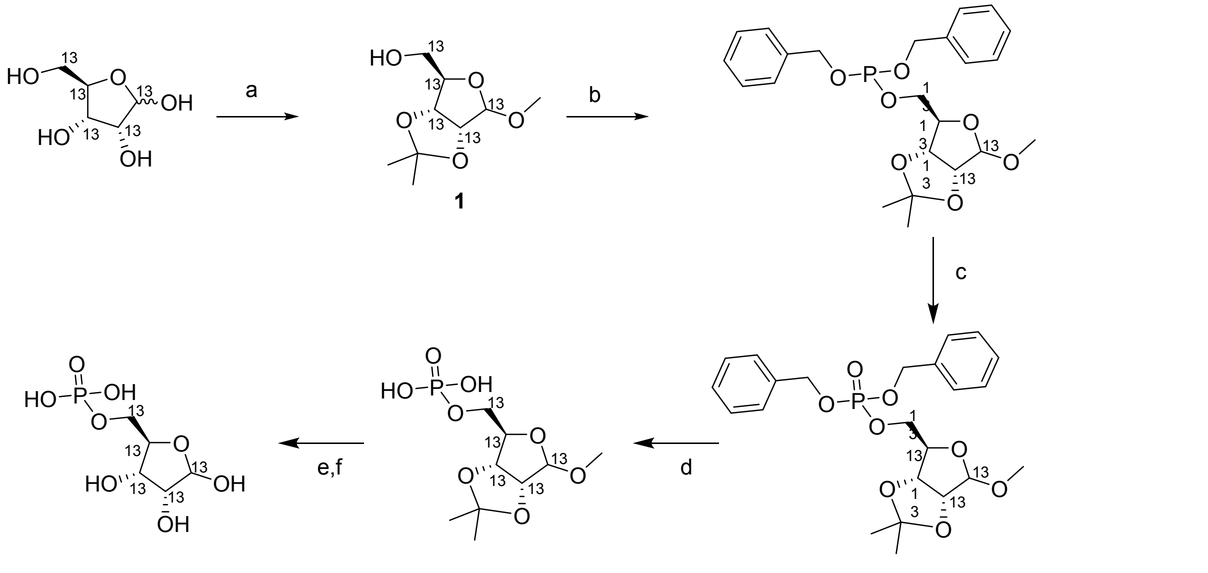

Scheme. a) Hydrochloric acid, acetone/methanol (1:1). b) 1*H*-tetrazole, dibenzyl *N*,*N*,diisopropylphosphoramidite, dichloromethane. c) *m*-CPBA, dichloromethane. d) H_2-Pd/C, methanol. e) Trifluoroacetic acid, water. f) Dowex® 50WX8 (Na^+ form).

Methyl-2,3-*O*-isopropylidene-β-D- (U-$^{13}C_5$) -ribofuranoside (1)

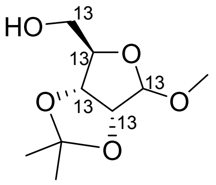

1

Concentrated hydrochloric acid catalytic (4 drops) was added to a suspension of isotope labelled ribose (965 mg, 6.43 mmol) in acetone (10 mL) and methanol (10 mL) at rt. The mixture was stirred for 36 h, neutralized with pyridine, and partitioned between water (10 mL) and ether (4 mL). The separated aqueous phase was extracted with ether (2 x 5 mL) and ethyl acetate (3 x 5 mL), and the combined organic phases were washed with saturated copper sulphate solution, water, and brine prior to drying and solvent evaporation. The residue was purified by column chromatography in hexane/ethyl acetate (1:1) rf: 0.51 to give 780 mg (60%) of a colourless oil.

^{1}H NMR (400 MHz, $CDCl_3$) δ (ppm): 4.83 (dm, J = 174 Hz, 1H, H_1), 4.65 (dm, J = 157 Hz, 1H, H_2), 4.45 (dm, J = 158 Hz, 1H, H_3), 4.23 (dm, J = 148 Hz, 1H, H_4), 3.44 (dm, J = 144 Hz, 1H, H_5), 3.3 (d, J = 4.5 Hz, 3H, $CH_{3\,OMe}$), 1.34 (s, 3H, $CH_{3\,Me}$), 1.28 (s, 3H, $CH_{3\,Me}$).

^{13}C NMR (101 MHz, $CDCl_3$) δ (ppm): 111.9 (s, $\underline{C}(CH_3)_2$), 109.6 (dd, J = 48.6, 1.2 Hz, C_1), 86.8 – 88.8 (m, C_4), 85.5 (ddd, J = 48.9, 31.0, 1.1 Hz, C_3), 79.1 – 82.5 (m, C_2) 63.6 (dd, J = 39.5, 3.0 Hz, C_5), 55.1 (dd, J = 3.9, 1.9 Hz, $CH_{3\,OMe}$), 26.2(t, J = 1.7 Hz, $CH_{3\,Me}$), 24.6 (S, $CH_{3\,Me}$).

Methyl-2,3-*O*-isopropylidene-β-D-(U-$^{13}C_5$)-ribofuranoside-5-dibenzylphosphate (2)

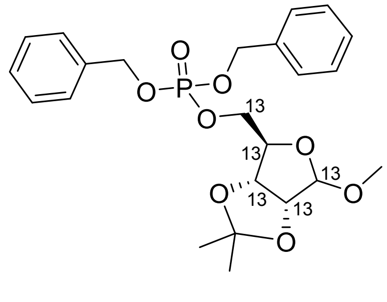

2

To a solution of compound **1** (780 mg, 3.82 mmol) and 1*H*-tetrazole (802 mg, 11.46 mmol) in CH_2Cl_2 (25 mL) was slowly added

dibenzyl (*N*,*N*-diisopropyl) phosphoramidite (2.57 mL, 7.64 mmol) at 0 ºC over a period of 10 min. The mixture was gradually warmed to room temperature, and stirred for 3 h. The mixture was then cooled to –40 ºC, and *m*-CPBA (1.58 g, 75% max., 13.38 mmol) was added. The mixture was allowed to warm in an ice bath over 1 h. The mixture was diluted with CH_2Cl_2, washed with saturated $Na_2S_2O_3$, $NaHCO_3$ and brine. The organic phase was dried over $MgSO_4$, filtered, and concentrated under reduced pressure. The crude product was purified by silica gel chromatography (EtOAc/hexane,1:5) to give compound **2** (1.24 g, 80%).

HRMS (ESI+) m/z: Calc. for $C_{18}{}^{13}C_5H_{30}O_8P$ $[M+H]^+$: 470.1841, found: 470.1844.

^{1}H NMR (400 MHz, CDCl$_3$) δ (ppm): 7.37 (s, 10H, H_{Arom}), 4.94 (dm, *J* = 172 Hz, 1H, H_1), 5.12 - 4.99 (m, 4H, $2CH_{2\ Bn}$), 4.62 (dm, *J* = 155, 1H, H_2), 4.52 (dm, *J* = 157 Hz, 1H, H_3), 4.31 (dm, *J* = 132 Hz, 1H, H_4), 3.96 (dm, J = 142 Hz, 2H, H_5), 3.27 (d, *J*=4.3, 3H, $CH_{3\ OMe}$), 1.48 (s, 3H, $CH_{3\ Me}$), 1.30 (s, 3H, $CH_{3\ Me}$).

^{13}C NMR (101 MHz, CDCl$_3$) δ (ppm): 128.6 (s, Arom), 128.0 (s, Arom), 112.5 (s, $\underline{C}(CH_3)_2$), 109.3 (d, *J* = 49.4 Hz, C_1), 85.4 – 84.0 (m, C_3, C_4), 82.4 – 80.4 (m, C_2), 69.5 (d, *J* = 5.6 Hz, $CH_{2\ Bn}$), 67.1 (dt, *J* = 43.2, 5.4 Hz, C_5), 55.0 (dd, *J* = 4.2, 2.0 Hz, $CH_{3\ OMe}$) 26.4 (s, $CH_{3\ Me}$), 24.9 (s, $CH_{3\ Me}$).

^{31}P NMR (162 MHz, CDCl$_3$) δ (ppm): 7.72 (s), -1.14 (dd, *J* = 8.7, 5.8 Hz).

Methyl-2,3-*O*-isopropylidene-D-(U-^{13}C$_5$)-ribofuranoside-5-phosphate (3)

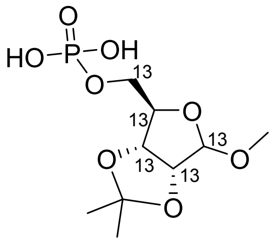

3

Compound **2** (726 mg, 1.56 mmol) dissolved in anhydrous MeOH (40 mL) and Pd/C 10% (130 mg) were stirred under H_2 for 2 h. The mixture was then filtered and concentrated to give compound **3** (430 mg, 97%).

HRMS (ESI+) m/z: Calc. for $C_4{}^{13}C_5H_{18}O_8P$ $[M+H]^+$: 290.0902, found: 290.0906

^{1}H NMR (400 MHz, MeOD) δ (ppm): 4.94 (dm, *J* = 175 Hz, 1H, H_1), 4.77 (dm, *J* = 156 Hz, 1H, H_2), 4.61 (dm, *J* = 158 Hz, 1H, H_3), 4.35 (dm, *J* = 137 Hz 1H, H_4), 3.94 (dm, *J* = 147 Hz, 2H, H_5), 3.27 (d, *J* = 4.3, 3H, $CH_{3\ OMe}$), 1.48 (s, 3H, $CH_{3\ Me}$), 1.30 (s, 3H, $CH_{3\ Me}$).

^{13}C NMR (101 MHz, MeOD) δ (ppm): 112.14 (s, $\underline{C}(CH_3)_2$), 109.31 (d, *J* = 49.3, $C_{1\beta}$), 108.37 (dd, *J* = 46.9, 3.0, $C_{1\alpha}$), 85.48 – 84.05 (m, $C_{4\beta}$, $C_{3\beta}$), 82.00 – 80.80 (m, $C_{2\beta,}$ C_α), 74.56 (dd, *J* = 47.0, 37.1, C_α), 71.72 – 70.72 (m, C_α), 67.76 – 67.10 (m, $C_{5\alpha}$), 66.15 (dt, *J* = 42.9, 5.1, $C_{5\beta}$), 53.91 (d, *J* = 2.4, $CH_{3\ OMe}$), 25.32 (s, $CH_{3\ Me}$), 23.64 (s, $CH_{3\ Me}$).

^{31}P NMR (162 MHz, MeOD) δ (ppm): 3.68 (s), -0.24 (s).

D-(U-^{13}C$_5$)-ribofuranoside-5-phosphate disodium salt (4)

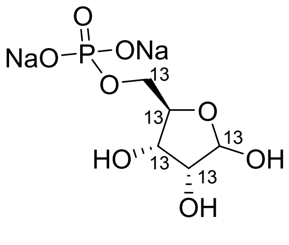

4

Compound **3** (430 mg, 1.51 mmol) was dissolved in water (1 mL) and trifluoroacetic acid was added to pH = 1. The solution was stirred at rt for 2 h and concentrated under diminished pressure, then the crude was purified through a Sep-Pak C_{18} cartridge. After the purification, the compound was eluted with water on an ion-exchange column of Dowex® 50WX8 (Na^+

form) and finally, the solvent was removed under diminished pressure to afford the compound **4** (300 mg, 87%).

HRMS (ESI+): Calc. for $^{13}C_5H_{12}O_8P$ = 236.0432, found = 236.0430.

^{13}C NMR (101 MHz, D_2O) δ (ppm): 100.92 (d, J = 45.8 Hz, $C_{1\beta}$), 96.26 (d, J = 40.6 Hz, $C_{1\alpha}$), 82.02 – 79.72 (m, $C_{4(\alpha+\beta)}$), 75.69 – 72.97 (m, $C_{2\beta}$), 72.01 – 68.27 (m, $C_{2\alpha}$, $C_{3(\alpha+\beta)}$), 67.43 – 64.37 (m, $C_{5(\alpha+\beta)}$).

^{31}P NMR (162 MHz, D_2O) δ (ppm): 0.47 (s).

Solid state NMR All experiments were carried out on Bruker AVANCE II 400 MHz wide bore spectrometers using 4 mm rotors. All extracellular matrix samples were packed into disposable inserts (Bruker) designed to be used inside these rotors. Cross-polarisation (CP) – magic-angle-spinning (MAS): Typical parameters: MAS frequency 10 kHz, ^{1}H 90° pulse 2.5 μs, contact time of 2.5 ms with ramped pulse on ^{1}H and square pulse on ^{13}C or ^{15}N at 70 kHz spin lock field strength, 70 kHz field strength SPINAL64 decoupling during acquisition, recycle delay 2s.

Single quantum-double quantum ^{13}C-^{13}C correlation experiments (SQ-DQ): Initial CP parameters as for ^{13}C CP experiments. At 10 kHz MAS, a 70 kHz POST-C7 pulse sequence[3] was applied on ^{13}C channel to excite double quantum coherence in 0.4 ms. Magnetisation was returned to zero quantum by another 0.4 ms of POST-C7 sequence. During double quantum evolution, 100 kHz Lee-Goldberg decoupling was applied on ^{1}H.

Proton-driven spin diffusion ^{13}C-^{13}C correlation experiments (PDSD): Initial CP parameters as for ^{13}C CP experiments. The magnetisation was allowed to evolve at single-quantum coherence during the incremental delay, and returned to zero quantum coherence by a ^{13}C 90° pulse (3.57 μs). ^{1}H decoupling was switched off during this mixing period (τ_M 20 ms and 100 ms in separate experiments), with a ^{13}C 90° readout pulse at the end of the mixing period. During both the incremented delay and acquisition periods, SPINAL64 decoupling was applied on ^{1}H at 70 kHz.

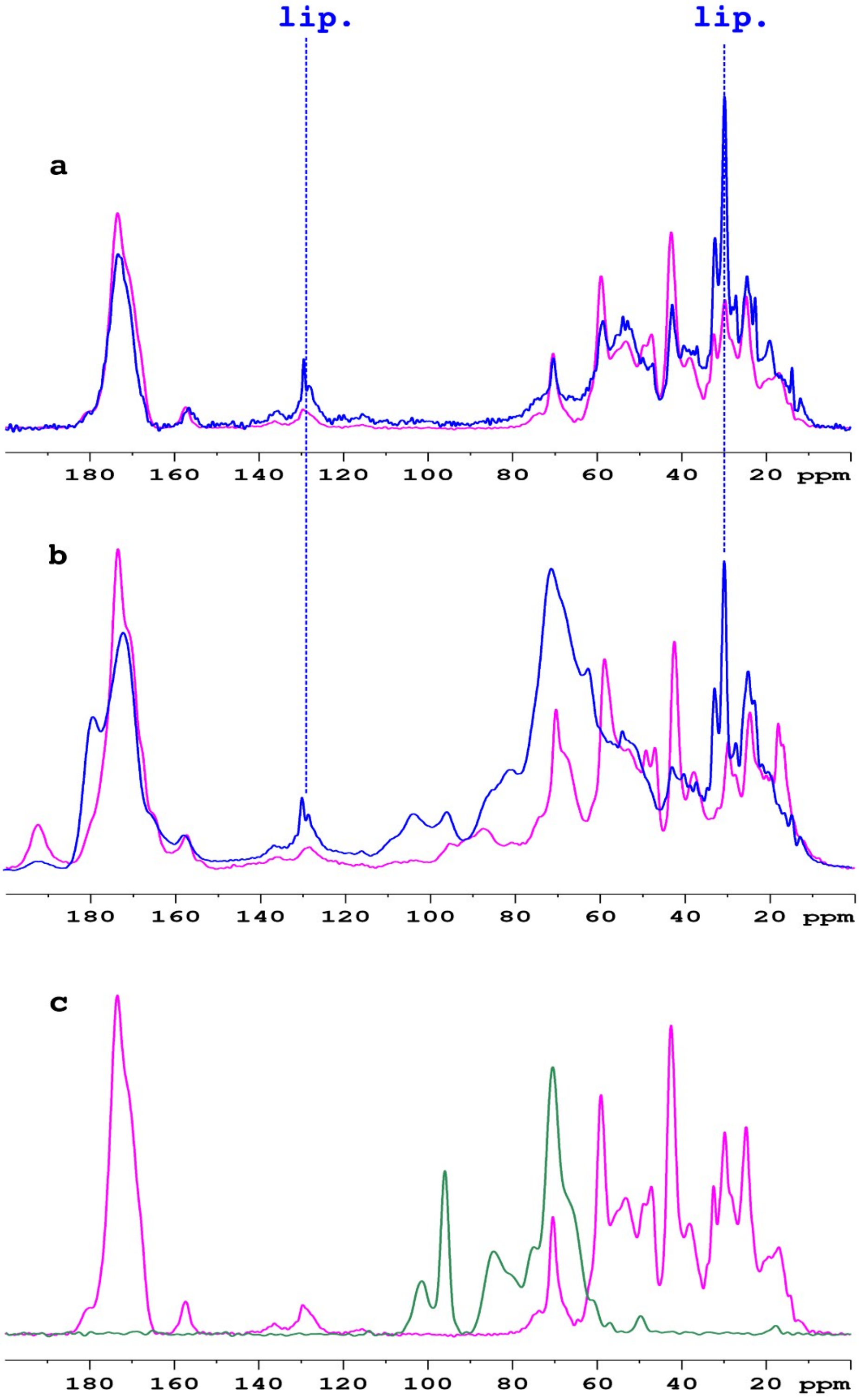

Figure S1. Figure S1. (a) and (b) Overlay of ^{13}C CP-MAS spectra of collagen (mauve) and VSMC ECM (blue). Lip. - Prominent lipid signals in VSMC ECM. (a) before incubation with U-$^{13}C_5$-R5P. (b) after incubation with U-$^{13}C_5$-R5P. (c) Overlay of spectra of pure collagen (mauve) and solid pure R5P, emphasizing the generation of new glycation product signals apparent in (a) and (b).

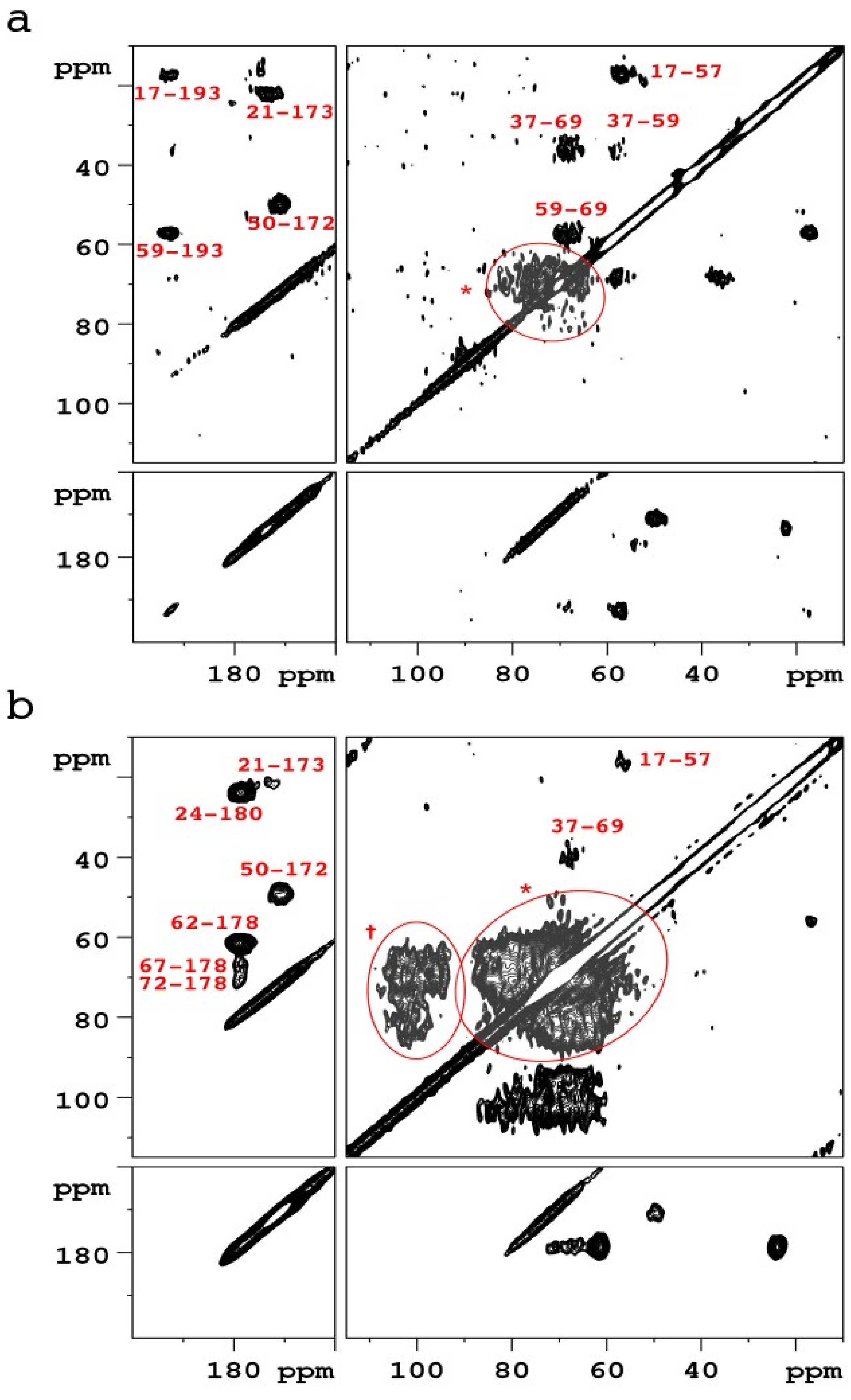

Figure S2. ^{13}C-^{13}C PDSD spectra of collagen (a) and VSMC ECM (b), reacted with U-$^{13}C_5$-R5P. * - Effects between vicinal diol carbons; †- Effects between hydroxyl carbons and (hemi)acetal and (hemi)aminal carbons.

References

1 W. Y. Chow, R. Rajan, K. H. Muller, D. G. Reid, J. N. Skepper, W. C. Wong, R. A. Brooks, M. Green, D. Bihan, R. W. Farndale, D. A. Slatter, C. M. Shanahan and M. J. Duer, *Science* 2014, **344**, 742.

2 P. T. Bullock, D. G. Reid, W. Y. Chow, W. P. Lau and M. J. Duer, *Bioscience reports*, 2014, **34**, 83.

3 M. Hohwy, H. J. Jakobsen, M. Eden, M. H. Levitt and N. C. Nielsen, *J. Chem. Phys.*, 1998, **108**, 2686.
